# Predictive Value of Routine Blood Test in patients with Early Esophageal Cancer: A Matched Case-Control Study

**DOI:** 10.7150/jca.56029

**Published:** 2021-06-05

**Authors:** Xiaoying Zhou, Han Chen, Weifeng Zhang, Xueliang Li, Xinmin Si, Guoxin Zhang

**Affiliations:** Department of Gastroenterology, First Affiliated hospital of Nanjing medical university.

**Keywords:** early esophageal cancer, MLR, PDW, inflammatory index

## Abstract

**Aims:** The present study was to evaluate the diagnostic value of routine blood test as potential inflammatory markers in early esophageal cancer (EEC) patients.

**Methods:** A matched case-control study was conducted by recruiting 314 patients who were pathologically diagnosed with EEC and then underwent Endoscopic Submucosal Dissection (ESD) from July 2015 to July 2019 in First Affiliated Hospital of Nanjing Medical University. Each EEC patient was matched against one healthy control on the criteria of gender, and age (±2 years). Additionally, a total of 40 subjects (20 cases and 20 controls) were also included in the validation set. Statistical analysis of selected hematological parameters was performed between the two groups. The correlation between preoperative blood indexes and clinicopathological characteristics after ESD in EEC patients were further assessed.

**Results:** Mono-factor analysis showed that the index of monocyte (*p<0.001*), MCV (*p=0.018*), MCH (*p=0.01*), MPV (*p=0.022*), PT (*p=0.003*), PT-INR (*p=0.003*), PDW (*p<0.001*) and MLR (*p<0.001*) were statistically significant in EEC patients when compared with those in healthy controls. Multivariate logistic regression analysis further identified that PDW and MLR was independently associated with the risk of early esophageal cancer (both *p<0.001*). The higher level of NLR *(P=0.007)* and MLR *(P=0.015)* were statistically significant with submucosal invasion in EEC patients and the level of MLR were significantly associated with larger tumor size *(P=0.030)*. The results of the validation group were in consistence with the primary group.

**Conclusions:** Hematological parameters of MLR and PDW can be used as an adjuvant tool for the diagnosis of EEC. Moreover, the value of MLR can reflect the invasion depth index.

## Introduction

Esophageal cancer is the 8^th^ most common cancer worldwide, and the 6^th^ most common cause of cancer-related death 1. There is significant variability in disease incidence by world region, with the highest rates occurring in Eastern Asia (17.0 per 100,000 in men, 5.4 per 100,000 in women) 2. In spite of the remarkable progress made during recent decades, still a small percentage of the patients with esophageal cancer suffer from recurrence or distant metastasis within 5 years due to diagnosis at advanced stage. Therefore, represented by the liquid biopsy (such as circulating tumor cells, circulating DNA, circulating miRNA, circulating lncRNA, and exosome), some newly developed diagnostic biomarker have been used to screen esophageal cancer at an early stage; however, their clinical use is still limited due to their uncertain role and high costs 3.

Recently, studies have shown that cancer-related systemic inflammation plays a significant role in the diagnosis and prognosis of cancers, especially in early cancers. Routine blood test (RBT) data are available to clinicians. The RBT measures the concentrations of white blood cells, red blood cells, platelets, platelet-to-lymphocyte ratio (PLR), neutrophil-to-lymphocyte ratio (NLR) and monocyte-to-lymphocyte ratio (MLR), aiming in the diagnosis of malignancies, inflammatory disease, and immune disorders 4. These indicators are believed to reflect inflammation, nutrition, and/or immunity and are reportedly associated with the prognosis in patients with esophageal cancer 5.

In this study, we aimed to address the diagnostic role of routine blood test (RBT) associated data in early esophageal cancer patients who perform ESD in our hospital. We compared the pre-operative data in these patients with healthy controls with colonic polyp who perform EMR with normal gastroscopy results.

## Materials and Methods

### Sample collection and ethics statement

Patients who were admitted to the First Affiliated Hospital of Nanjing Medical University to perform ESD for the first time from 2015 to 2019 and pathologically diagnosed as early stage esophageal cancer were enrolled in this study. Inclusion criteria were listed as follows: (1) histopathological diagnosis of early esophageal cancer on Endoscopically-resected specimens; (2) pT1 stage carcinoma (no tumor invasion beyond the submucosa); Exclusion criteria: (1) histopathological diagnosis of esophageal adenocarcinoma or other types of esophageal cancer; (2) mixed type of esophageal cancer; (3) tumor with the undefined pathological origin and metastatic esophageal cancer; (4) no history of previous malignancies and anticancer therapies; (5) patients younger than 18 years; (6) perioperative mortality; (7) distant metastases; (8) previous medical history of hematologic or rheumatic autoimmune disease; (9) acute or chronic infections during inpatient stays; (10) a previous history of taking aspirin or warfarin. The controls were colonic polyp who performed EMR with normal gastroscopy results. Data regarding patients' demographics and laboratory values were retrospectively reviewed through hospital medical database records. None of the subjects in the control group had any recorded history of malignancies and were matched to the cases in terms of age and sex. The clinical characteristics and laboratory data of the study population are summarized in Tables 1 and 2. Ethics approval for the use of human subjects was obtained from the Ethics Committee of the first affiliated hospital of Nanjing medical University. We also recruited 40 patients (20 early esophageal cancer patients and 20 healthy controls) from the first affiliated hospital of Soochow university for external validation.

### Blood assessment

Blood values had been taken into consideration at the time of diagnosis before administration of any treatment when patients and controls were admitted to our hospital. Venous blood specimens were drawn into sterile standard tubes containing ethylene diamine tetraacetic acid (EDTA) as an anticoagulant and evaluated within 1 h after venipuncture using a Beckman Coulter UniCel DxH800 hematology analyzer. The Beckman Coulter UniCel® DxH 800 was used for analyzing routine blood markers including White Blood Cell (WBC), neutrophils, monocytes, lymphocytes, platelet (PLT), Red Blood Cell (RBC), Hemoglobin; hematocrit (HCT), Platelet Distribution Width (PDW), Mean Platelet Volume (MPV), Mean corpuscular volume (MCV), Mean corpuscular hemoglobin (MCH) and Mean corpuscular hemoglobin concentration (MCHC). Inflammatory markers of Neutrophil-Lymphocyte Ratio (NLR), Platelet-Lymphocyte Ratio (PLR), and Monocyte-to-Lymphocyte Ratio (MLR) were calculated subsequently. The Sysmex® CS-5100 hemostasis system was applied for coagulation analysis of fibrin (FIB), Activated Partial Thromboplastin Time (APTT), Thrombin Time (TT), Prothrombin Time (PT), and D-dimer. The Beckman Coulter AU5800 Clinical Chemistry Analyzer was used for assessing lactate dehydrogenase (LDH) and retinol-binding proteins (RBP). The Roche® Cobas e602 module was used for tumor markers of Alpha-Fetoprotein (AFP), Carcinoembryonic Antigen (CEA) and Cancer antigen 19-9 (CA199).

### Statistical analysis

The statistical analyses were performed by using SPSS version 23.0 (SPSS, Inc. Chicago, IL, USA) and data are presented as median with Interquartile Range (Q). Normality test were applied by Shapiro-Wilk and Kolmogorov-Smirnov test. Data with normal distribution were considered if *p* value less than 0.05. For comparisons, the t test (2-tailed) were applied for data with normal distribution while Mann-Whitney U test were performed in data with abnormal distribution. Receiver-operating characteristics (ROC) curve analysis was further performed to identify optimum cut-off values of selected hematological parameters. Multi-variant logistic regression analysis was further conducted to identity the independent risk factors of EEC. P value less than 0.05 was considered statistically significant.

## Results

### Comparisons between early esophageal cancer group and control group

**Figure 1:**
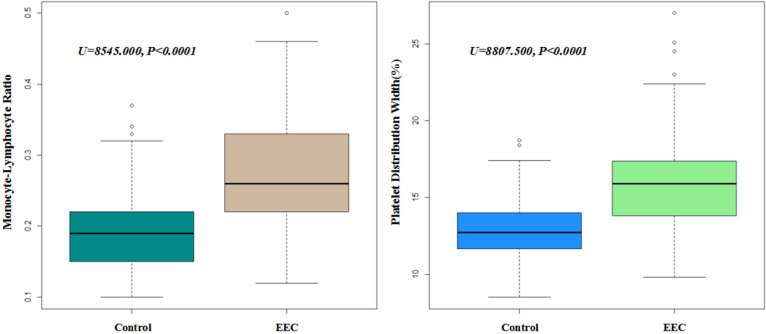
** The expression levels of PDW and MLR in EEC and control group (P<0.001).** Boxplots of MLR and PDW in both EEC and control group.

A total of 314 early esophageal cancer patients from 2015 to 2019 to perform ESD and 329 healthy individuals were enrolled into this retrospective study. After matching by age (+/- 2 years old) and gender, 219 pairs were finally enrolled to compare the levels of blood parameters. The laboratory blood parameters of early esophageal cancer patients and controls were summarized in Table 1. Shapiro-Wilk test showed that all the data of blood test were with abnormal distributions (all p<0.001). Thus, Mann-Whitney U test was performed and identified that the levels of monocyte (*p<0.001*), MCV (*p=0.018*), MCH (*p=0.01*), MPV (*p=0.022*), PT (*p=0.003*), PT-INR (*p=0.003*), PDW (*p<0.001*) and MLR (*p<0.001*) in the early esophageal cancer patients were significantly higher than those in the control group, while the levels of lymphocyte (*p<0.001*) and RBC (*p=0.003*) were significantly lower in the early esophageal cancer patients. Furthermore, we selected these significant factors to further perform multivariate regression analysis and we found that PDW and MLR were significantly higher in early esophageal cancer patients (*all p<0.001*) (Figure 1).

**Figure 2:**
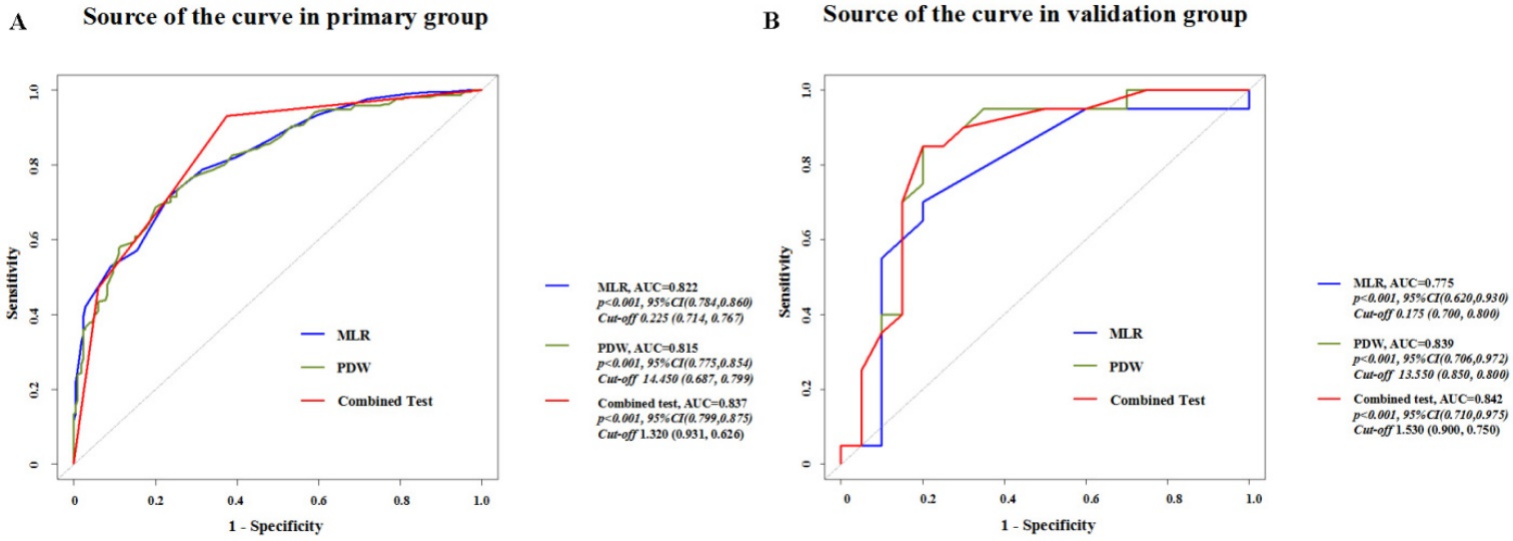
ROC curve analysis of PDW, MLR and combined test in primary group **(A)** and validation group **(B)**.

ROC curve analysis showed that the AUCs of PDW and MLR were 0.815 (95% CI: 0.775-0.854) and 0.822 (95% CI: 0.784-0.86), respectively. The AUC of combined diagnosis of both PDW and MLR was 0.837 (95% CI: 0.799-0.875). The sensitivity of PDW was 0.687 and its specificity was 0.799. The sensitivity of MLR was 0.717 and its specificity was 0.77. The combined sensitivity of combined diagnosis was 0.931 and the specificity was 0.626. According to DeLong's test, there is no significant difference of ROC between PDW (Z=-1.0398, P=0.2985) or MLR (Z=-1.0778, P=0.2811) alone with PDW and MLR combined, however, combined PDW and MLR diagnosis was recommended because of the higher sensitivity (Figure 2A).

We also set a new group (n=40, with 20 EECs and 20 controls) from another medical center for external validation. After validation, AUCs of PDW and MLR were 0.839 (95% CI: 0.706-0.972) and 0.775 (95% CI: 0.620-0.930), respectively. The AUC of combined diagnosis of both PDW and MLR was 0.842 (95% CI: 0.710-0.975) (Figure 2B).

### Assess the correlation between clinical characteristics of early esophageal cancer patients with hematological parameters

The data of 314 patients with early esophageal cancer were further analyzed according to their clinical characteristics, including tumor size, tumor location, histologic grade, invasion depth, lymphovascular invasion. As shown in Table 2, we found that the NLR and MLR values in invasion to submucosal group were significantly higher than that in invasion to intramucosal group (*p=0.007* and *p=0.015*, respectively). The MLR value in patients with tumor size ≥2 (cm) was significantly higher than in those with tumor size <2 (cm) (*p=0.03*). There was no significant difference in MLR, RBC and CEA in each subgroup.

## Discussion

In this retrospective study, we explored the significance of pretreatment hematological parameters in the diagnosis of early esophageal cancer who went to our hospital to perform ESD. To the best of our knowledge, this has not been reported in the literature so far. Although this is a retrospective study, it is also a real world evidence study. In order to minimize the selection bias, we performed a matched case-control study, that is we matched patients' age and sex when we select enrolled patients in the two groups, in order to eliminate their influence on routine blood tests. Furthermore, we also set a validation group from another hospital to further confirm our results. The results are in validation is consistent with primary group.

Hematological parameters in the noninvasive routine blood test have long been considered as markers for systemic inflammatory response 6. Neutrophils play a role in tumor angiogenesis through the production of proangiogenic factors, which contribute to the adhesion and seeding of distant sites 7. In addition, neutrophilia can restrain the immune system by inhibiting the cytolytic effects of immune cells 8. Platelets, which can be recruited by tumor cells, can protect tumor cells from the immune reaction and facilitate their dissemination 9. In contrast, lymphocytes, as the basic component of the adaptive and innate immune system, are essential in providing antitumor immunity. Specifically, CD4+ and CD8+ T cells recognize tumor antigens and have been proven to induce tumor cell apoptosis 10. Since it has been recognized that there are complex interactions between tumors and inflammatory responses, research is increasingly focusing on the use of inflammation biomarkers to predict the behavior of tumors. Higher MLR, NLR and PLR are associated with poorer prognosis 11. In our study, we showed that PDW and MLR were significantly higher in EEC patients compared to the control group. NLR and MLR exhibited positive correlations with invasion depth in preoperative EEC patients. PLR exhibited positive correlations with larger tumor size. However, studies on the diagnostic value of inflammation biomarkers for early esophageal cancer are inadequate.

There were several possible mechanisms. Tumor-associated macrophages (TAMs), which are derived from circulating monocytic precursors, also play key roles in the inflammatory microenvironment of tumor progression. TAM can stimulate tumor cell proliferation, promote angiogenesis, and favor invasion and metastasis by producing growth and angiogenic factors, as well as protease enzymes, which degrade the extracellular matrix 12. Lymphocytes, different from neutrophils and monocytes, play a crucial role in host cell-mediated immunity regulation, which is important to destruct residual malignant cells and related micrometastases. It is now widely believed that tumor-infiltrating lymphocytes (TILs) are associated with better clinical outcomes in cancers 13. Consequently, higher MLR has a significant diagnostic value of EEC.

The possible cause by which PDW have an effect on cancer progression is that platelets facilitate the hypercoagulability in tumor. Platelets take part in the different steps of angiogenesis including proliferation, migration, extracellular matrix degradation, and adhesion of endothelial cells 14. Activated platelets are involved at cancer-associated thrombosis by releasing inflammatory information, and interacting with neutrophils and monocytes. The PDW that is one of the platelet indices not merely check platelet volume heterogeneity, but also reactive platelet activity 15. Recently, several studies revealed that a high PDW is an unfavorable prognosis factor in melanoma patients, laryngeal cancer, and gastric cancer 16-18. Bone marrow cells malfunction may be associated with the lower PDW. PDW reflects platelet heterogeneity, which is caused by heterogeneous demarcation of megakaryocytes. Cytokines, including interleukin-6 (IL-6), macrophage colony stimulating factor (M-CSF), and granulocytes colony stimulating factor (G-CSF), have an effect on megakaryocytic maturation, platelet production, and platelet size. IL-6 facilitates cancer cell proliferation, invasion, and metastasis. IL-6 is correlated with the prognosis and depression of cancer patients and is considered to the therapy target. Moreover, G-CSF stimulates megakaryopoiesis and constrains tumor to proliferation. M-CSF was an important factor in the cancer microenvironment, involving in the interactions between tumor-infiltrated macrophages and tumor cells 19. Those reports are in accord with the point that activated platelets participate in the pathogenesis of esophageal cancer.

## Limitations

However, limitations regarding the application of MLR and PDW in clinical practice still remain. Firstly, although we found statistically significant differences between the tumor groups regarding MLR and PDW, the ROC analyses were not ideal, and the AUCs were not as high as expected. Using the Youden index, the sensitivities and specificities of the cut-off values were not ideal either, which hampers the clinical applications of our results. For an accurate diagnosis of early esophageal cancer, using MLR or PDW alone is not sufficient; thus, we recommend an integrated and combined approach using a variety of methods. Secondly, the lymph node metastasis status can only be got from surgery. So, the predictive value of routine blood test index for the lymph node metastasis of these patients after ESD need to be further identified. Further research on MLR and PDW and its interactions with early esophageal cancer or other early gastrointestinal cancers are expected.

## Conclusions

The routine blood test is the most accessible and fundamental examination, which has long been proposed as an essential assistant tool for disease diagnosis. Our results showed that MLR and PDW can be effective in distinguishing early esophageal cancer patients from healthy individuals. Furthermore, MLR is an effective index in evaluating tumor invasion depth. Our results need to be verified by large-scale clinical studies with follow-up study. However, to the best of our knowledge, this study is the first to provide comprehensive insights into hematological parameters of routine blood testing in early esophageal cancer patients. More prospective studies in the future are warrant to perform such as the diagnostic value of RBT combined with the liquid biopsy (such as circulating tumor cells, circulating DNA, circulating miRNA, circulating lncRNA, and exosome), which may be more efficient to diagnose early esophageal cancer.

## Figures and Tables

**Table 1:** Baseline characteristics of hematological parameters of the EEC patients and controls

Hematological Parameters	EEC (n=219), M (Q)	Control (n=219), M (Q)	*P*-value
Age (Year)	63 (10)	62 (12)	*.573*
Gender (male, N%)	140 (63.9%)	140 (63.9%)	*1.000*
WBC (×10^9^/L)	5.43 (1.87)	5.39 (2.11)	*.209*
MO (×10^9^/L)	0.43 (0.16)	0.33 (0.15)	<.0001*
LY (×10^9^/L)	1.55 (0.61)	1.75 (0.68)	<.0001*
NE (×10^9^/L)	3.14 (1.51)	3.10 (1.45)	*.282*
PLT (×10^9^/L)	186 (72)	191 (68)	.050*
NLR	1.86 (1.20)	1.79 (0.79)	*.251*
PLR	112.39 (43.84)	110.77 (46.53)	*.934*
MLR	0.27 (0.12)	0.19 (0.07)	<.0001*
EO (×10^9^/L)	0.12 (0.12)	0.12 (0.10)	*.679*
BA (×10^9^/L)	0.02 (0.03)	0.02 (0.02)	*.857*
RBC (×10^12^/L)	4.49 (0.64)	4.56 (0.62)	.003*
HGB (g/L)	137.00 (19.30)	139.00 (20.00)	*.094*
HCT (%)	40.50 (6.30)	40.80 (7.50)	*.280*
MCV (fL)	91.70 (5.10)	90.00 (5.30)	.018*
MCH (pg)	30.70 (1.70)	30.40 (1.90)	.010*
MCHC (g/L)	335.00 (12.00)	334.00 (11.00)	*.475*
RDWCV (%)	12.80 (1.10)	12.90 (1.00)	*.934*
PCT (%)	0.20 (0.07)	0.21 (0.07)	*.248*
MPV (fL)	11.20 (1.72)	10.90 (1.60)	.022*
PDW (%)	15.80 (3.70)	12.70 (2.40)	<.0001*
FIB (g/L)	2.50 (0.79)	2.47 (0.8)	*.952*
PT (s)	11.70 (0.80)	11.60 (0.80)	.003*
PTINR	1.02 (0.07)	1.01 (0.07)	.003*
APTT (s)	27.65 (3.70)	28.10 (2.70)	*.648*
TT (s)	18.20 (1.10)	18.30 (1.20)	*.460*
DD2 (mg/L)	0.22 (0.22)	0.22 (0.22)	*.361*
AFP (ng/ml)	2.86 (1.62)	2.82 (1.92)	*.816*
CEA (U/ml)	2.24 (1.78)	2.11 (1.54)	*.153*
CA199 (U/ml)	9.22 (9.18)	9.25 (8.72)	*.828*
CYFRA211 (ng/ml)	1.74 (0.93)	1.89 (1.19)	*.214*
NSE (ng/ml)	16.88 (6.90)	17.28 (7.58)	*.161*

**Note:** Abbreviations: EEC, Early Esophageal Cancer; WBC, White Blood Cell; MO, Monocyte; LY, Lymphocyte; NE, Neutrophils; PLT, platelet; NLR, Neutrophil-Lymphocyte Ratio; PLR, Platelet-Lymphocyte Ratio; MLR, Monocyte-to-Lymphocyte Ratio; EO, Eosinophils; BA, Basophils; RBC, Red Blood Cell; HGB, Hemoglobin; HCT, hematocrit; MCV, Mean Corpuscular Volume; MCH, Mean Corpuscular Hemoglobin; PDW, Platelet Distribution Width; MPV, Mean Platelet Volume; FIB, fibrin; PT, Prothrombin Time; INR, International Normalized Ratio; APTT, Activated Partial Thromboplastin Time; TT, Thrombin Time; DD2, D-dimer; AFP, Alpha-Fetoprotein; CEA, Carcinoembryonic Antigen; CA199, Cancer antigen 19-9; CYFRA211, cytokeratin 19 fragment; NSE, Neuron Specific Enolase.

**Table 2:** Association of significant routine blood test index and clinical pathological characteristics of EEC patients underwent ESD

Variable	No. of case	NLR		PLR		MLR		RBC (×10^12^/L)		PDW (%)		CEA (U/ml)	
		Median (Q)	*P*-value	Median (Q)	*P*-value	Median (Q)	*P-value*	Median (Q)	*P*-value	Median (Q)	*P*-value	Median (Q)	*P*-value
**Tumor size**													
≥2 (cm)	115	2.03(1.32)	*.199*	116.63(57.26)	*.086*	0.28(0.13)	.030*	4.40(0.68)	*.250*	15.85(4.18)	*.090*	2.19(1.72)	*.870*
<2 (cm)	187	1.82(1.12)		106.15(42.92)		0.24(0.12)		4.51(0.62)		15.20(3.50)		2.24(1.73)	
**Tumor location**													
Upper thoracic	30	1.85(1.13)	*.574*	109.77(52.99)	*.743*	0.25(0.09)	*.604*	4.51(0.65)	*.859*	15.10(3.14)	*.078*	1.84(1.14)	*.420*
Middle thoracic	89	1.82(1.06)		110.12(50.8)		0.26(0.13)		4.52(0.64)		15.70(4.90)		2.22(1.89)	
Lower thoracic	171	1.98(1.20)		110.00(45.42)		0.27(0.12)		4.44(0.63)		15.40(3.70)		2.36(1.67)	
Histologic grade													
Carcinoma *in situ*	217	1.81(1.10)	*.065*	108.00(43.08)	*.293*	0.25(0.13)	*.149*	4.47(0.65)	*.828*	15.70(3.77)	*.908*	2.24(1.75)	*.017*
Well	66	2.10(1.26)		119.47(59.17)		0.29(0.14)		4.46(0.73)		14.65(3.65)		2.30(1.55)	
Moderately	12	1.74(1.21)		104.32(35.46)		0.27(0.13)		4.42(0.84)		15.30(2.90)		2.22(1.68)	
**Invasion Depth**													
Intramucosal, pT1a	235	1.81(1.02)	.007*	109.89(43.99)	*.487*	0.25(0.11)	.015*	4.48(0.64)	*.925*	15.70(3.87)	*.215*	2.16(1.62)	*.299*
Submucosal, pT1b	60	2.50(1.55)		112.49(61.77)		0.301(0.15)		4.39(0.68)		14.70(3.40)		2.60(2.09)	
LV Invasion													
Positive	15	2.47(1.63)	*.259*	108.00(35.18)	*.894*	0.28(0.1)	*.088*	4.50(0.95)	*.697*	14.50(3.09)	*.293*	2.76(2.14)	*.475*
Negative	281	1.86(1.13)		110.12(46.11)		0.26(0.13)		4.47(0.64)		15.50(3.80)		2.24(1.70)	
